# Genomic Analysis of Rotavirus G8P[8] Strains Detected in the United States Through Active Surveillance, 2016–2017

**DOI:** 10.3390/v17091230

**Published:** 2025-09-09

**Authors:** Mary C. Casey-Moore, Mathew D. Esona, Slavica Mijatovic-Rustempasic, Jose Jaimes, Rashi Gautam, Mary E. Wikswo, John V. Williams, Natasha Halasa, James D. Chappell, Daniel C. Payne, Mary Allen Staat, Geoffrey A. Weinberg, Michael D. Bowen

**Affiliations:** 1 Centers for Disease Control and Prevention, Atlanta, GA 30329, USAhsr7@cdc.gov (S.M.-R.); ogg3@cdc.gov (J.J.); ijs0@cdc.gov (R.G.);; 2 University of Pittsburgh Medical Center, Children’s Hospital of Pittsburgh, Pittsburgh, PA 15224, USA; jvwilliams2@wisc.edu; 3 Vanderbilt University Medical Center, Nashville, TN 37232, USA; natasha.halasa@vumc.org (N.H.);; 4 Division of Infectious Diseases, Cincinnati Children’s Hospital Medical Center, Cincinnati, OH 45229, USA; daniel.payne@cchmc.org (D.C.P.); mary.staat@cchmc.org (M.A.S.); 5 Department of Pediatrics, University of Cincinnati College of Medicine, Cincinnati, OH 45229, USA; 6 Department of Pediatrics, University of Rochester School of Medicine and Dentistry, Rochester, NY 14642, USA; geoff_weinberg@urmc.rochester.edu

**Keywords:** rotavirus, RVA, G8P[8], USA, NVSN

## Abstract

G8 rotaviruses are primarily associated with animals and infrequently cause infections in humans. The first detection of G8 strains in humans occurred around 1979, and since then, their presence has been sporadic, particularly in the United States (U.S.). During the 2016–2017 rotavirus surveillance season, the New Vaccine Surveillance Network (NVSN) identified 36 G8P[8] rotavirus strains across four sites in the U.S. This study presents the whole-genome characterization of these G8P[8] strains, along with comparative sequence analyses against the current vaccine strains, Rotarix and RotaTeq. Each strain exhibited a DS-1-like backbone with a consensus genotype constellation of G8P[8]-I2-R2-C2-M2-A2-N2-T2-E2-H2 and exhibited high genetic similarities to G8P[8] strains previously detected in Europe and Asia. Clinical analysis revealed no significant differences in hospitalization rates, length of stay, or severity scores between G8P[8] RVA-positive and non-G8P[8] RVA-positive subjects. Additionally, phylodynamic analysis determined the evolutionary rates and the most recent common ancestor for these strains, highlighting the importance of ongoing monitoring of rotavirus genotypes to assess the spread of these emerging G8P[8] strains.

## 1. Introduction

Group A rotavirus (RVA) remains a significant cause of severe diarrheal diseases in children under five years old worldwide, with an estimated 108,470 deaths in 2021. RVA accounts for approximately 24% of diarrheal deaths, highlighting the need for continued focus on vaccination efforts and improved access to effective treatments, particularly in high-mortality settings where the impact of rotavirus remains substantial [1]. To mitigate this burden, the World Health Organization recommends the integration of rotavirus vaccines into global immunization programs, including Rotarix^®^ (GlaxoSmithKline, Rixensart, Belgium) and RotaTeq^®^ (Merck & Co., West Point, PA, USA) [2]. Currently, 86 countries administer Rotarix as part of their national immunization programs, while 33 countries use RotaTeq, and 14 countries, including the United States (U.S.), utilize both vaccines [3]. In the U.S., vaccine coverage has reached 77%, with vaccine effectiveness (VE) against hospitalization and emergency department visits reported at 83% for Rotarix and 84% for RotaTeq [4].

The RVA genome is composed of 11 double-stranded RNA segments (dsRNA), which encode the viral proteins (VP1 to VP4, VP6, and VP7) and non-structural proteins (NSP1 to NSP5/6) [5]. Using the extended classification system, the genotype constellation for VP7-VP4-VP6-VP1-VP2-VP3-NSP1-NSP2-NSP3-NSP4-NSP5/6 of the RVA strain can be described using the abbreviations Gx-P[x]-Ix-Rx-Cx-Mx-Ax-Nx-Tx-Ex-Hx, respectively [6]. Currently, there are 42 G, 58 P, 32 I, 28 R, 24 C, 24 M, 39 A, 28 N, 28 T, 32 E, and 28 H genotypes identified for human and animal hosts [7].

There are six predominant G and P genotype combinations in humans, classified into two main genogroups: Wa-like (Gx-P[x]-I1-R1-C1-M1-A1-N1-T1-E1-H1) or genotype 1 constellation and DS-1-like (Gx-P[x]-I2-R2-C2-M2-A2-N2-T2-E2-H2) or genotype 2 constellation. The most common RVA genotype combinations are G1P[8], G2P[4], G3P[8], G4P[8], G9P[8], and G12P[8], with G1P[8], G3P[8], G4P[8], G9P[8], and G12P[8] typically having a Wa-like genotype constellation, while G2P[4] carries a DS-1-like constellation [8]. In the U.S., post-vaccine surveillance from 2007 to 2013 indicated a shift in genotype predominance, moving from G1P[8] to G3P[8] in 2009, and then G3P[8] to G12P[8] in 2012 [9,10]. Notably, genotype G8, which is more prevalent in bovines, rarely causes rotavirus infections in humans [11,12]. However, instances of G8 strains leading to human infections, especially those with the VP4 genotype P[8] and a DS-1-like genetic backbone, have sporadically emerged in Africa, Asia, Central Europe, and South America between 2003 and 2022 [13,14,15,16,17,18,19,20,21,22,23,24].

The emergence of new rotavirus genotypes in the post-vaccine era underscores the importance of ongoing surveillance to monitor circulating strains. Since 2006, the New Vaccine Surveillance Network (NVSN) has been actively conducting surveillance in the U.S. for acute gastroenteritis (AGE) related to rotavirus [25]. During the 2016–2017 RVA surveillance season, there was an increased detection of G8P[8] strains at four NVSN sites: Cincinnati Children’s Hospital Medical Center (Cincinnati, Ohio), Vanderbilt University Medical Center (Nashville, Tennessee), University of Pittsburgh Medical Center Children’s Hospital of Pittsburgh (Pittsburgh, Pennsylvania), and University of Rochester School of Medicine and Dentistry (Rochester, New York). To better understand these emerging strains, we first analyzed the clinical characteristics of the G8P[8] subjects in comparison to other RVA-positive cases detected during the surveillance season. We performed whole-genome sequencing to examine the evolutionary relationships and genetic makeup of these strains, comparing the genetic profiles of the emerging G8P[8] strains with those of wild-type RVA and vaccine strains. Additionally, we conducted phylodynamic analyses to estimate the evolutionary rates and the time to the most recent common ancestor (tMRCA) of the G8P[8] strains, providing further insights into their epidemiological trends and genetic dynamics.

## 2. Materials and Methods

### 2.1. The Study Enrollment

Subjects were enrolled at NVSN surveillance sites while visiting the emergency department (ED) or hospitalized with AGE (≥3 diarrhea episodes and/or ≥1 vomiting episode within 24 h) between December 2016 and November 2017. Children enrolled in the ED but hospitalized within 6 days for the same illness were categorized as inpatients. Healthy controls (HC) were enrolled at each site’s general pediatric outpatient clinic(s) and matched to enrolled AGE subjects based on age group during a scheduled well-visit that occurred within 14 days of AGE subjects’ enrollment. AGE stools were collected within 10 days of symptom onset, and HC stools within 5 days of enrollment. Stool samples were refrigerated and processed within 24 h, then frozen at −80 °C. Families provided demographic and clinical data. Rotavirus vaccination status was verified through official records.

### 2.2. Sample Selection for Whole Genome Analysis

Centers for Disease Control and Prevention (CDC) received 328 presumptive RVA-positive stool samples, identified by enzyme immunoassays, from AGE and HC subjects during the 2016–2017 NVSN season. 11.6% (38 out of 328) were RVA-negative by real-time RT-PCR (qRT-PCR) testing. With the remaining 290 RVA positive samples, Sanger sequencing was performed to determine VP7 and VP4 genotypes [26]. Among the genotyped samples, 35 AGE subjects and 1 HC were confirmed as G8P[8]. Samples with cycle threshold values >25 during the NSP3 qRT-PCR were excluded, leaving 29 samples (28 AGE and 1 HC) for next-generation sequencing (NGS) analysis.

### 2.3. Viral dsRNA Extraction for NGS

To extract viral RNA, 10% stool suspensions were prepared using phosphate-buffered saline and RNA was extracted from the suspension using the MagNA Pure Compact RNA Isolation Kit on the automated MagNA Pure Compact Instrument (Roche Applied Science, Indianapolis, IN, USA), as described previously [27]. cDNA library synthesis, amplification, and NGS: Sequencing templates and libraries were prepared using the NEBNext Ultra RNA Library Prep Kit for Illumina v1.2 and NEBNext Multiplex Oligos for Illumina (New England Biolabs, Ipswich, MA, USA) according to the manufacturer’s specifications. NGS was carried out on an Illumina MiSeq sequencer using the MiSeq reagent kit v.2 with 500 cycles, and the standard 250 bp paired-end reads method.

### 2.4. Whole-Genome Sequence Analysis

Contigs were assembled from individual reads by reference-guided assembly with default parameters using CLC Genomics Workbench 24.0 software (http://www.clcbio.com/products/clc-genomics-workbench/ (accessed on 8 March 2021)). Genotypes were determined according to the guidelines of the Rotavirus Classification Working Group (RCWG) (https://rega.kuleuven.be/cev/viralmetagenomics/virus-classification/rcwg (accessed on 31 May 2024)) [28]. The assembled sequences were compared with sequences of known RVA genotypes using NCBI’s BLASTN tool (https://blast.ncbi.nlm.nih.gov/Blast.cgi (accessed on 4 June 2024)) or the Bacterial and Viral Bioinformatics Resource Center (BV-BRC) Subspecies Classification tool (https://www.bv-brc.org/ (accessed on 4 June 2024)) [29,30].

### 2.5. Phylogenetic, Sequence, and Structural Analyses

Alignments and comparative analysis of the full-length sequences for each gene segment were performed as previously described [31]. Multiple sequence alignments were made using the MUSCLE algorithm implemented in MEGA 6 software (https://www.megasoftware.net/ (accessed on 10 June 2024)) [32]. Amino acid substitutions were categorized as ‘radical’ by referencing the BLOSUM62 matrix scores and changes in the resulting amino acid properties (https://www.ebi.ac.uk/Tools/psa/emboss_needle/ (accessed on 10 June 2024)). Nucleotide and amino acid sequence identities among strains were calculated for each gene based on distance matrices prepared using the *p*-distance algorithm in MEGA 6 software. The DNA Model Test program within CLC Genomics Workbench 24.0 software [33] was used to identify optimal evolutionary models that best fit sequence datasets using Corrected Akaike Information Criterion (AICc). Maximum-likelihood (ML) trees were constructed using CLC Genomics Workbench 24.0 software with 1000 bootstrap replicates to estimate branch support. Each ML tree was visualized and edited using MEGA 6 software.

### 2.6. Clinical Severity Score

To examine clinical severity of AGE, we used the Modified Vesikari Severity Score (MVSS). We categorized the 20-point MVSS into 3 groups: mild (score 0–10), moderately severe (score 11–15), and very severe (score 16–20) disease [34].

### 2.7. Bayesian Phylodynamic Analyses

The evolutionary rate and tMRCA were determined through the Bayesian Markov Chain Monte-Carlo (MCMC) approach implemented in BEAST v.10.5.0 [35]. The dataset included a total of 79 VP7 and 61 VP4 sequences obtained from 1965 to 2019 and 2000–2019, respectively. Both genes were analyzed using an HKY nucleotide substitution model, strict clock model and CTMC Scale Reference Prior model [36]. VP7 phylodynamic analysis was run with discrete gamma-distributed rate heterogeneity model [37]. Five and seven independent MCMC analyses were run for 100 million generations and diagnosed using Tracer software v1.7.2 (http://tree.bio.ed.ac.uk/software/tracer (accessed on 15 May 2025)), for VP7 and VP4, respectively, to achieve an effective sample size (ESS) of >200. The maximum clade credibility tree (MCCT) was annotated using TreeAnnotator and viewed in FigTree v1.4.4 (http://tree.bio.ed.ac.uk/software/figtree (accessed on 15 May 2025)). Evolutionary rates were evaluated using Tracer, and mean value with the 95% highest posterior density (95% HPD) interval was reported.

## 3. Results

Within the 2016–2017 surveillance season, 328 presumptive RVA-positive stool specimens were submitted from the NVSN surveillance sites to CDC for genotype identification. Of those specimens, 290 (88.4%) were confirmed positive for RVA and successfully assigned VP7 and VP4 genotypes using Sanger sequencing.

### 3.1. VP7 and VP4 Genotypes

**Figure 1:**
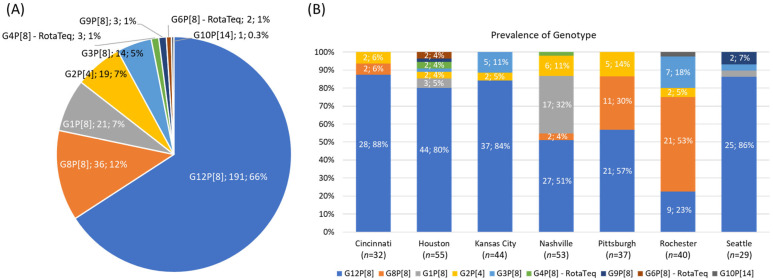
The cumulative RVA genotyping results (**A**) and the genotyping results differentiated by surveillance site (**B**) for RVA-positive typeable specimens collected from the New Vaccine Surveillance Network (NVSN) 2016–2017 season. For (**B**), the numbers and percentages are displayed for genotypes detected more than once.

The RVA genotyping results from the VP7 and VP4 genes (Figure 1A) featured: 191 G12P[8] strains (66%), 36 G8P[8] strains (12%), 21 G1P[8] strains (7%), 19 G2P[4] strains (7%), 14 G3P[8] strains (5%), 3 G4P[8] RotaTeq vaccine strains (1%), 3 G9P[8] strains (1%), 2 G6P[8] RotaTeq vaccine strains (1%), and 1 G10P[14] strain (0.3%).

Figure 1B shows the prevalences of genotypes differentiated by surveillance site. G12P[8] was the most commonly detected strain in all sites, except for Rochester, where 21 strains (53%) were identified as G8P[8], with the second most common being G12P[8] (9 strains, 23%). Pittsburgh also exhibited a significant portion of G8P[8] strains, with 11 strains (30%). Cincinnati and Nashville each had reported 2 G8P[8] strains. Notably, Nashville had a large portion of G1P[8] strains, accounting for 32% of the total strains at that site. Across the other surveillance sites, the remaining genotypes were detected at lower frequencies.

### 3.2. Clinical Observations

Given the high volume of G8P[8] strains identified, we investigated the clinical aspects of these G8P[8] cases compared to the other RVA-positive specimens obtained during the 2016–2017 NVSN season, which included all 35 subjects with G8P[8] strain detection (excluding the HC) (Table 1). The age distribution of subjects ranged from 0 to over 60 months, with a median age of 23 months (IQR: 14–38 months). The age distribution of G8P[8] positive subjects was comparable to that of non-G8P[8] positive subjects, with no significant differences observed (*p* = 0.2934). The highest proportion of cases in both groups was in the 12–23-month age range, accounting for 31% of G8P[8] cases and 28% of non-G8P[8] cases.

Vaccination rates were comparable between the groups: 49% of G8P[8] positive subjects received RotaTeq compared to 41% of non-G8P[8] positive subjects, while 23% of G8P[8] positive subjects received Rotarix versus 16% of non-G8P[8] positive subjects. Additionally, 20% of G8P[8] positive subjects were unvaccinated, compared to 32% in the non-G8P[8] group. Hospitalization rates among G8P[8] positive subjects mirrored that of non-G8P[8] positive subjects, with approximately half of cases being managed as outpatients and half requiring inpatient care. The median length of stay for hospitalized subjects was also comparable: 1 day [IQR 1, 2] for G8P[8] positive and 2 days [IQR 1, 3] for non-G8P[8] positive patients. No significant difference in clinical severity was observed between groups, with median MVSS of 13 [IQR 10, 15] for both G8P[8] positive and non-G8P[8] positive AGE subjects.

### 3.3. G8P[8] Whole-Genome Analysis

To investigate the genetic characteristics of the G8P[8] strains, we conducted whole-genome sequencing on 29 study strains using NGS techniques. Of these strains, two were from Cincinnati, 11 from Pittsburgh (including 1 strain from a HC, 3000820368) and the remaining 16 strains from Rochester. Seven strains were not included in the analysis due to low sample titers.

**Figure 2:**
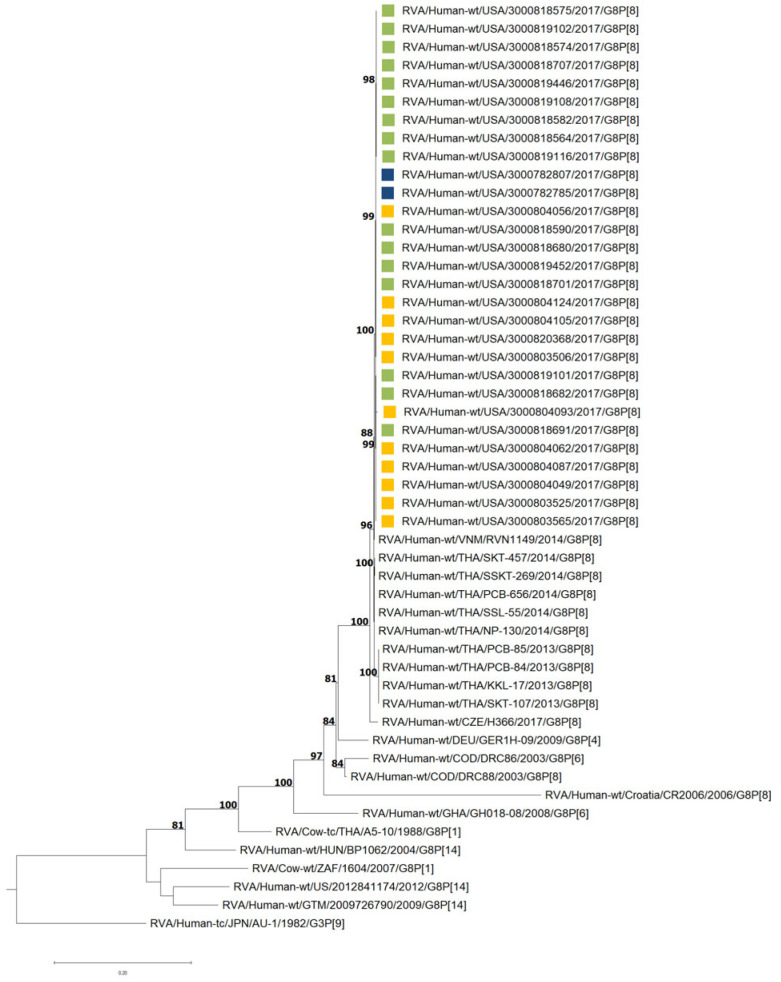
Maximum likelihood phylogram revealing the genetic relatedness of the concatenated, whole-genome ORF sequences for the 29 G8P[8] NVSN strains. The strains are color-coded according to their surveillance site locations: green for Rochester, navy for Cincinnati, and yellow for Pittsburgh. Bootstrap values ≥ 70% are indicated at branch nodes where applicable and the scale bar indicates the number of nucleotide substitutions per site.

All strains possessed a DS-1-like backbone with a consensus genotype constellation of G8-P[8]-I2-R2-C2-M2-A2-N2-T2-E2-H2. The genetic relatedness among the 29 G8P[8] study strains was assessed through the construction of a concatenated phylogenetic tree using whole-genome ORF sequences. As shown in Figure 2, the tree reveals a high degree of genetic similarity among the study strains, regardless of their site location. Notably, the study strains are closely related to G8P[8] strains previously identified in Vietnam [17], Thailand [16], and the Czech Republic [23].

### 3.4. VP7 and VP4 Phylogenetic Analyses and Sequence Identity

**Figure 3:**
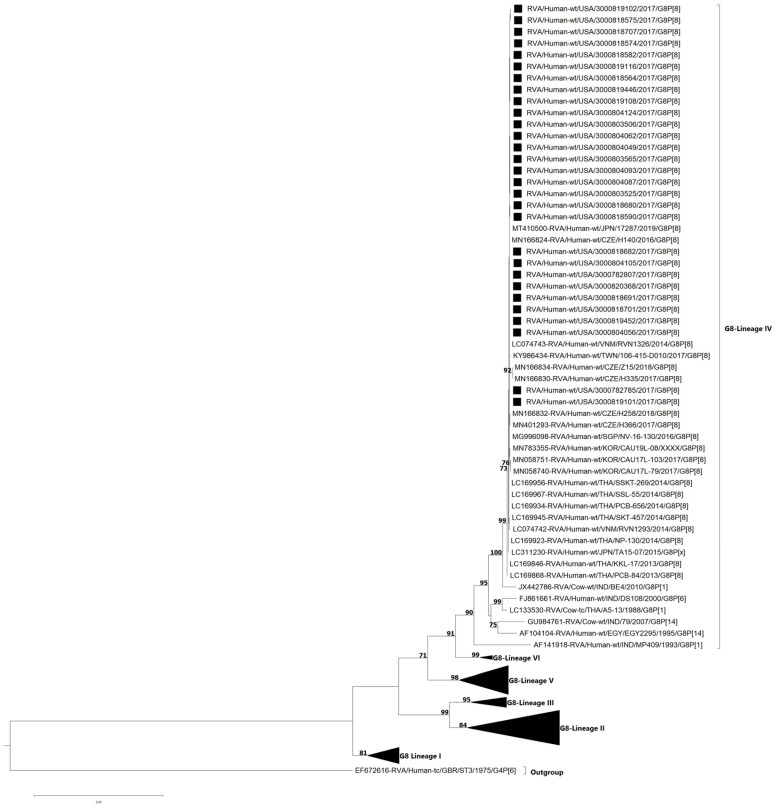
Maximum likelihood phylogenetic tree illustrating the genetic relationships among the ORF nucleotide sequences of the G8 VP7 gene for 2016–2017 NVSN strains. The GTR+G+T evolutionary model was used for phylogenetic inference. NVSN G8P[8] study strains are indicated with a black square. Only bootstrap values ≥ 70% are shown adjacent to each branch node. Scale bar indicates the number of nucleotide substitutions per site.

The VP7 and VP4 genes of the G8P[8] study strains were subjected to phylogenetic and sequence identity analyses. VP7 genes of G8 strains separated into six lineages (I–VI) [16,23]. All 29 study strains clustered in lineage IV and were most closely related to G8P[8] DS-1-like strains that have emerged from Japan [15,38], Czech Republic [23], Vietnam [17], Taiwan, Singapore [39], South Korea, and Thailand [16] (Figure 3). The nucleotide (nt) and amino acid (aa) identities of the study strains amongst themselves ranged from 99.7 to 100% and 99.4–100%, respectively (Table S1), while the nt (aa) similarities of the NVSN study strains compared to lineage IV GenBank strains were 90.0–100% (92.9–100%). In contrast, the NVSN study strains were genetically less similar to strains from other G8 lineages, corresponding to nt (aa) identities of 83.2–84.7% (92.4–95.4%) with lineage I, 84.1–86.0% (92.0–94.4%) with lineage II, 83.8–86.1% (92.9–97.1%) with lineage III, 84.6–87.7% (91.6–95.7%) with lineage V, and 89.3–89.8% (95.0–95.4%) with lineage VI (Table S1).

**Figure 4:**
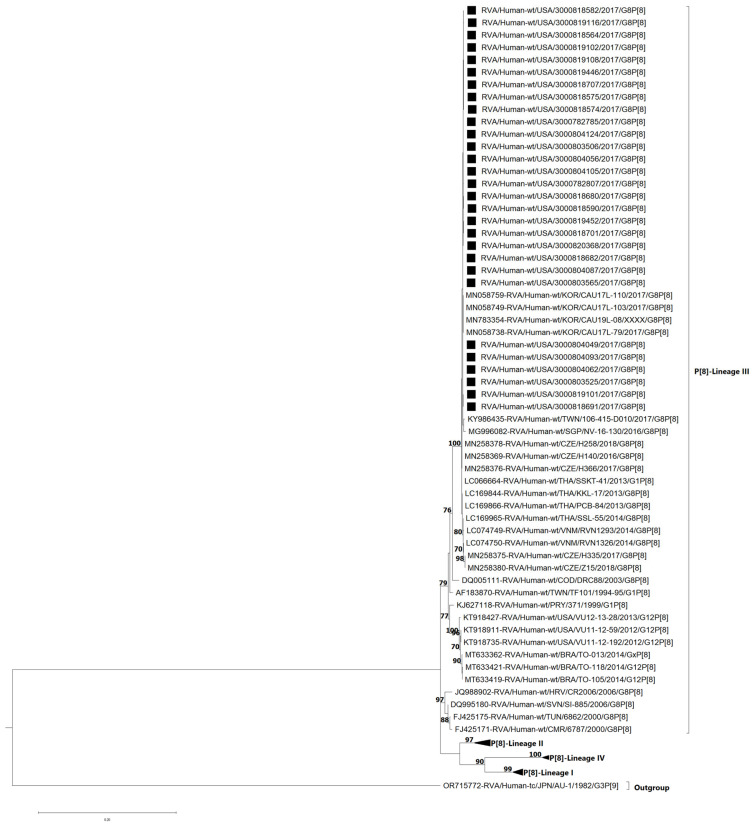
Maximum likelihood phylogenetic tree illustrating the genetic relationships among the ORF nucleotide sequences of the P[8] VP4 gene for 2016–2017 NVSN strains. The GTR+G+T evolutionary model was used for phylogenetic inference. NVSN G8P[8] study strains are indicated with a black square. Lineages are indicated in roman numerals. Only bootstrap values ≥70% are shown adjacent to each branch node. Scale bar indicates the number of nucleotide substitutions per site.

The VP4 genes of P[8] are divided into four established lineages (I–IV) [16,23] (Figure 4). All of the G8P[8] study strains clustered within lineage III (bootstrap = 100%). Lineage III consists of previously described DS-1-like G8P[8] strains that have emerged in Europe and Asia [15,16,17,23,39], as well as a strain from the Democratic Republic of Congo [13] detected in 2003. The nt (aa) identities of NVSN study strains amongst themselves were 99.8–100% (99.5–100) (Table S2). NVSN study strains shared nt (aa) identities ranging from 90.2 to 90.6% (93.8–94.3%) with lineage I, 92.4–97.7% (95.2–96.8%) with lineage II, 93.5–100% (93.9–100%). with lineage III and 88.1–88.6% (89.7–92.9%) with lineage IV (Table S2). Full phylogenetic lineage trees for G8 VP7 and P[8] VP4 are provided in Supplementary Figures S1 and S2, respectively.

### 3.5. Phylogenetic Analyses and Sequence Identity of Internal Genes

The phylogenetic and sequence identity analyses for the internal genes can be found in Figures S3–S11 and Table S3, respectively. The VP6, VP1, VP2, and VP3 genes of all NVSN study strains clustered with gene sequences of DS-1-like strains belonging to genotypes I2, R2, C2, and M2, respectively (Figures S1–S4). These strains exhibited high genetic similarities to the previously described G8P[8] strains detected in Europe and Asia [20,21,22,34,36]. Interestingly, the NVSN study strains also shared close relation to G2P[4] strains circulating in the U.S. from 2011 to 2015, except for VP2. The nt (aa) identities among NVSN study strains and GenBank strains ranged from 85.5 to 100% (96.5–100%), 85.6–99.8% (97.0–99.8%), 85.3–99.8% (96.9–100%), and 82.9–99.9% (89.6–100%) for VP6, VP1, VP2, and VP3, respectively (Table S3).

The NSP1, NSP2, NSP3, NSP4, and NSP5 genes of all NVSN study strains were assigned to the A2, N2, T2, E2, and H2 genotypes, respectively (Figures S7–S11). Results of phylogenetic analyses paralleled findings made previously for the internal genes. The NVSN study strains clustered with cognate gene sequences of DS-1-like strains belonging to genotype 2 and exhibited the closest genetic relationship with previously reported G8P[8] of European and Asia origin, followed by G2P[4] strains. The nt (aa) identities among NVSN study strains and GenBank strains of their corresponding genotype ranged from 92.3 to 100% (91.9–100%), 86.5–99.9% (94.0–100%), 90.2–100% (94.2–100%), 87.5–100% (94.9–100%), and 95.5–100% (96.5–100%) for NSP1, NSP2, NSP3, NSP4, and NSP5, respectively (Table S3).

### 3.6. Comparative Analysis of VP7 Antigenic Epitopes with Reference Strains

**Figure 5:**
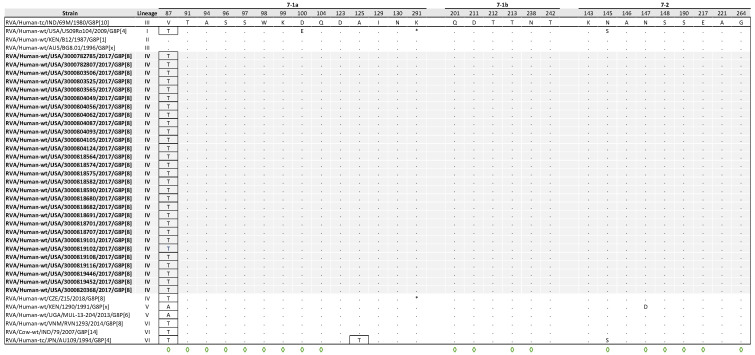
Alignment of antigenic residues in VP7 between G8 strain 69M, NVSN strains, and GenBank strains representing the six G8 lineages. Antigenic residues are divided into three epitopes (7-1a, 7-1b, and 7-2). NVSN strains are colored gray throughout the alignment for visibility. Period (.) indicates that the amino acid is conserved. Asterisk (*) indicates the absences of aa in partial sequences. Radical amino acid substitutions are boxed. Amino acid sites that have been shown to escape neutralization with monoclonal antibodies are indicated with a green diamond.

The VP7 gene contains three structurally defined antigenic epitopes, 7-1a, 7-1b, and 7-2 regions, which together consist of 29 aa residues [40]. Of the 29 aa positions, 19 have been identified as capable of evading neutralization by monoclonal antibodies due to various mutations [41]. A comparative analysis of the antigenic epitopes between the reference strain 69M and the NVSN study strains revealed a single conserved substitution (V87T) in region 7-1a. V87T is a radical change located in a neutralization escape site (Figure 5).

### 3.7. Comparative Analysis of VP4 Antigenic Epitopes with Vaccine Strains

**Figure 6:**
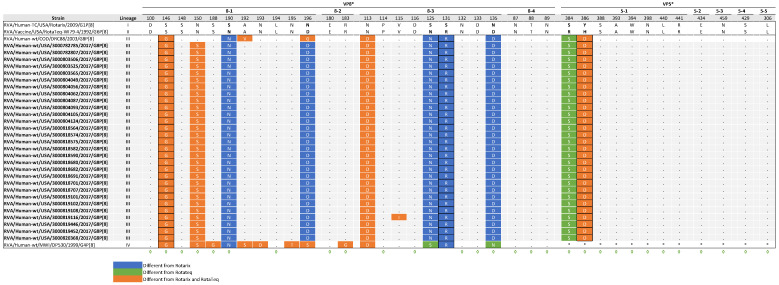
Alignment of antigenic residues in VP4 between vaccine strains, NVSN strains, and GenBank strains representing the four P[8] lineages. Antigenic residues are divided in three antigenic epitopes in VP8* and five antigenic epitopes in VP5*. Amino acids that differ between Rotarix and RotaTeq are indicated in boldface. NVSN and GenBank residues that differ from Rotarix and RotaTeq are colored blue and green, respectively. Residues colored in orange are different from both Rotarix and RotaTeq. Period (.) indicates that the amino acid is conserved. Asterisk (*) indicates the absences of aa in partial sequences. Radical amino acid substitutions are boxed. Amino acid sites that have been shown to escape neutralization with monoclonal antibodies are indicated with a green diamond.

The VP4 protein forms spikes on the surface of the virus and plays a crucial role in the attachment and entry of the virion into host cells. The VP8 subunit, which constitutes the head of VP4, contains four surface-exposed epitopes (8-1 to 8-4) made up of 25 amino acids. In contrast, VP5*, which forms the body of VP4, has five epitopes (5-1 to 5-5) consisting of 12 amino acids. Antibodies targeting the VP8 epitopes have been shown to neutralize rotavirus infection by preventing viral attachment, while antibodies against the VP5 epitopes can block virion penetration of the host cell membrane [42,43]. Of the 37 aa residues spanning these epitopes, 29 are known neutralization escape mutation sites [44,45,46,47]. Comparative analysis of the antigenic epitopes identified considerable differences when comparing Rotarix and RotaTeq strains to NVSN study strains (Figure 6). Specifically, the NVSN study strains contained 9 to 10 residues that differed within the P[8] epitopes of Rotarix and 5 residues that differed within the P[8] epitopes of RotaTeq. The amino acid differences were in the VP8* 8-1 and 8-3, and VP5* 5-1 epitopes. VP8* 8-2 and 8-4 and VP5* 5-2 through 5-5 epitopes were highly conserved compared to the vaccine strains.

Comparing the NVSN study strains to both vaccine strains revealed a shared radical substitution (S146G) in VP8* 8-1, along with an additional radical substitution in VP5* 5-1 at amino acid residue 386 relative to Rotarix (Y386D) and RotaTeq (H386D). When looking at differences only from Rotarix, there was one common radical substitution (S131R) in VP8* 8-3. S146G, Y386D, and H386D are located within neutralization escape sites. The VP4 antigenic epitopes of 3000819116 displayed an additional mutation (V115I) within VP8* 8-3.

### 3.8. VP7 and VP4 Bayesian Evolutionary Analyses

Phylodynamic analyses were conducted using a dataset comprising 79 G8 nucleotide sequences from the VP7 gene and 61 P[8] nucleotide sequences from the VP4 gene. We initially planned to utilize the same sequences as in the phylogenetic analysis for the subsequent BEAST analysis; however, the ESS parameters were found to be below 200. To address this, we incorporated additional G8 sequences for the VP7 analysis, while concentrating exclusively on P[8]-lineage III strains for the VP4 analysis. These adjustments in the phylodynamic analyses resulted in ESS values exceeding 200.

**Figure 7:**
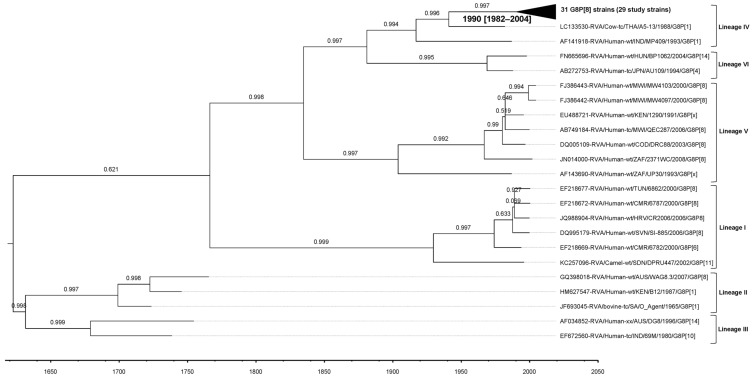
Maximum Clade Credibility Tree (MCCT) for the VP7 gene, constructed using Bayesian Markov chain Monte-Carlo (MCMC) analysis based on 79 G8 strains. Lineages are indicated on the right-hand side of the tree. Each strain is labeled with its name and year of isolation, while a time scale is provided below the tree. The horizontal purple bars at the phylogenetic nodes represent the 95% credible interval for the estimated age of each respective node.

For the G8-lineage IV strains in our study, the estimated evolutionary rate was 8.73 × 10^−4^ (standard deviation 6.65 × 10^−6^; 95% HPD interval [6.73 × 10^−4^, 1.08 × 10^−3^]) nt substitutions/site/year. The tMRCA was estimated to be 1990 (95% HPD interval 1982–2004) (Figure 7).

**Figure 8:**
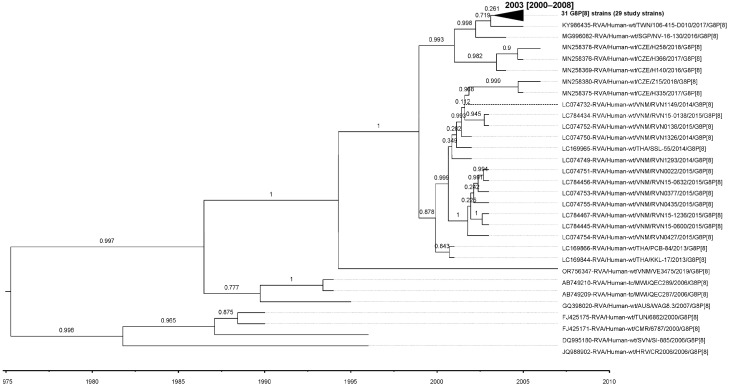
MCCT for the VP4 gene, constructed using Bayesian MCMC analysis based on 61 P[8] lineage III strains. Each strain is labeled with its name and year of isolation, while a time scale is provided below the tree. The horizontal purple bars at the phylogenetic nodes represent the 95% credible interval for the estimated age of each respective node.

Similarly, the evolutionary rate for the P[8]-lineage III strains analyzed in this study was estimated at 8.23 × 10^−4^ (standard deviation 5.28 × 10^−6^; 95% HPD interval [5.69 × 10^−4^, 1.07 × 10^−3^]) nt substitutions/site/year, with a tMRCA of 2003 (95% HPD interval 2000–2008) (Figure 8).

## 4. Discussion

Our study sequenced 29 of the 36 G8P[8] RVA strains collected during the 2016–2017 NVSN season and found that all carried a DS-1 like genetic backbone. Historically, G8 strains have been rarely detected in the U.S., with sporadic reports including one adult with G8P[14] in 2012 [48] and three children with G8P[4] in 2019 [49]. Interestingly, during the subsequent 2017–2018 NVSN season, five G8P[8] strains were identified out of 133 RVA-positive cases, but surprisingly, no G8P[8] strains were detected in the following NVSN seasons (2018–2019, 2019–2020, and 2020–2021), highlighting the transient nature of this strain’s presence in the U.S. rotavirus landscape (unpublished data) [50].

Phylogenetic analysis of NVSN study G8 VP7 and P[8] VP4 segments revealed that the bovine–human DS-1-like strains clustered with strains described in Japan, Thailand, Vietnam, Czech Republic, and Singapore [15,16,17,23,39]. The internal genes also revealed a close genetic relationship with G8P[8] strains from these regions. The high extent of nucleotide and amino acid identity indicates significant genetic conservation among these strains, suggesting that the emergence of G8P[8] strains in the U.S. was likely due to the introduction of strains from Europe and Asia, rather than the evolution of strains already circulating within the four NVSN surveillance regions. The genetic homogeneity of the complete genome sequences observed in this study further supports the possibility that this introduction may have resulted from a single introduction event. Further investigation is required to understand their origin and the details of geographical spread.

Interestingly, all G8P[8] samples detected in our study were from patients with AGE, underscoring the clinical relevance of these strains circulating in the population. In our clinical observations, we found no significant differences in hospitalization rates or severity scores between G8P[8] positive and non-G8P[8] positive subjects. The high proportion of vaccinated G8P[8] positive subjects in our study supports the effectiveness of rotavirus vaccines in mitigating disease severity against these emerging strains. Additionally, one G8P[8] strain included in the NGS analysis was derived from a HC, potentially representing an asymptomatic infection. Ongoing studies are crucial to understand vaccine-mediated protection against G8P[8].

While we identified antigenic disparities between the NVSN study and vaccine strains by comparing the VP4 antigenic epitopes, the clinical characteristics of G8P[8] infections do not support a significant role for immune escape from the immunity elicited by the two available vaccines. The comparison of the VP7 ORF from the NVSN study strains to the first identified G8 strain, 69 M, revealed a high level of conservation among antigenic epitopes, with only one radical mutation, V87T, within epitope 7-1a. This mutation was conserved among the NVSN study strains and observed in GenBank strains representing various G8 lineages (I, IV, and VI). This high level of conservation suggests that while certain mutations may mediate escape from neutralization, the overall structure of epitopes remains stable across these G8P[8] strains. The presence of 87T in Rotarix and three of the five RotaTeq strains indicates that existing vaccines may still offer adequate protection against these circulating strains.

It has been proposed that changes in RVA neutralization epitopes could drive immune-escape and ultimately influence VE [51]. In 2017, Hoque et al. analyzed a G8P[8] outbreak in Japan to assess infection severity and VE for Rotarix and RotaTeq [14]. Their study compared G8P[8] with other RVA strains detected during the outbreak and examined the differences between G8P[8] strains found in vaccinated and unvaccinated children with AGE. The analysis found no notable differences in clinical characteristics between G8P[8] and other RVA strains. Notably, the attack rates of G8P[8] strains were similar in children regardless of vaccination status, while the VE against moderate and severe G8P[8] infections was found to be strong. These findings suggest that although vaccination does not provide complete protection against G8P[8] strains, it plays a pivotal role in reducing the severity [14]. Our findings align with those reported by Hoque et al. underscoring that vaccination continues to mitigate disease severity even in the context of emerging G8P[8] strains.

Evolutionary rates for RNA viruses typically range from 1 × 10^−3^ to 1 × 10^−6^ substitutions/site/year, with RVA VP7 and VP4 segments often evolving at the higher end of this spectrum [52]. For VP7, previous phylodynamic analyses by Degiuseppe et al. estimated the evolutionary rate of G8-lineage IV strains at 3.7 × 10^−3^ substitutions/site/year (95% HPD: 1.4 × 10^−3^–8.2 × 10^−3^), with a tMRCA around 1986 (95% HPD: 1984–1988) [53]. In the present study, the estimated rate for G8-lineage IV was 8.73 × 10^−4^ substitutions/site/year (95% HPD: 6.73 × 10^−4^–1.08 × 10^−3^), with a tMRCA of 1990 (95% HPD: 1982–2004). While our rate estimate is lower than that reported by Degiuseppe et al., it remains within the broad ranges observed for RVA VP7 genotypes, and our tMRCA estimate is consistent with previously reported timelines.

Published estimates of VP4 P[8] evolutionary rates also vary by lineage. For example, OP354-like P[8] strains sampled between 1988 and 2012 were estimated to evolve at 1.30 × 10^−3^ substitutions/site/year (95% HPD: 0.97–1.69 × 10^−3^), with a tMRCA of 1987 (95% HPD: 1985–1988) [54]. Broader G9P[8] datasets yielded a rate of 0.87 × 10^−3^ (95% HPD: 0.75–1.00 × 10^−3^) [55]. In comparison, the rate estimated for P[8]-lineage III strains in the present study was 8.23 × 10^−4^ substitutions/site/year (95% HPD: 5.69 × 10^−4^–1.07 × 10^−3^), which falls within the published range and indicates consistency with the evolutionary dynamics of other circulating P[8] strains. The tMRCA for our dataset was estimated at 2003 (95% HPD: 2000–2008), concordant with studies suggesting that P[8] lineages emerged in the late 1990s to early 2000s. Taken together, these comparisons demonstrate that the evolutionary rates and divergence times estimated in our study are broadly consistent with those reported in the literature for both VP7 G8-lineage IV and VP4 P[8]-lineage III strains.

Despite these insights, this study has limitations. First, samples with low viral titers were excluded from NGS analysis, which reduced the number of G8P[8] strains available for genomic characterization. Of the 36 confirmed G8P[8] cases, only 29 samples were sequenced, potentially limiting the breadth of genetic insights obtained. Second, the relatively small number of G8P[8] cases (*n* = 35) restricted our ability to perform robust comparisons of clinical characteristics between G8P[8]-positive subjects and those infected with non-G8P[8] rotavirus strains. This limited sample size may have reduced our ability to detect significant differences, highlighting the need for larger studies to validate these findings. Finally, to achieve adequate ESS values in BEAST, we expanded the VP7 dataset to include additional G8-lineage IV strains and restricted the VP4 analysis to P[8]-lineage III strains. While these adjustments ensured robust phylodynamic outputs, they may limit the generalizability of the evolutionary rate and tMRCA estimates across all G8 and P[8] strains.

In conclusion, the G8P[8] strains detected in the U.S. during the 2016–2017 season were genetically similar to strains circulating in Europe and Asia, suggesting introduction events rather than local evolution. Despite antigenic differences compared with vaccine strains, vaccination appeared to reduce disease severity. Phylodynamic analyses showed evolutionary rates and divergence times consistent with prior reports, underscoring the robustness of these findings. Continued genomic and clinical surveillance will be essential for understanding the molecular epidemiology and evolution of emerging RVA strains.

## Figures and Tables

**Table 1:** Clinical characteristics of the enrolled subjects with acute gastroenteritis.

	RVA Positive (*n* = 253)	G8P[8] Positive (*n* = 35)	Non-G8P[8] Positive (*n* = 218)	*p*-Value ^a^
**Age Group**	**No. (%)**			
0–11 months	54 (21%)	3 (9%)	51 (23%)	
12–23 months	73 (29%)	11 (31%)	62 (28%)	
24–35 months	55 (22%)	9 (26%)	46 (21%)	
36–47 months	32 (13%)	5 (14%)	27 (12%)	
48–59 months	10 (4%)	3 (9%)	7 (3%)	
60+ months	29 (11%)	4 (11%)	25 (11%)	
Age in months, median (IQR)	23 (14–38)	23 (13–38)	25 (16–41)	0.2934
**Vaccination Characteristics**	**No. (%)**			
Eligible patients ^b^	243 (96%)	35 (100%)	208 (96.4%)	
*Product*				
Unvaccinated	74 (30%)	7 (20%)	67 (32%)	
Rotarix (partial or complete series)	42 (17%)	8 (23%)	34 (16%)	
RotaTeq (partial or complete series)	102 (42%)	17 (49%)	85 (41%)	
Mixed	12 (5%)	2 (6%)	10 (5%)	
Unknown series	13 (5%)	1 (3%)	12 (6%)	
**Hospitalization Status**	**No. (%)**			
ED/outpatient	127 (50%)	17 (49%)	110 (50%)	
Hospitalized/inpatient	126 (50%)	18 (51%)	108 (50%)	
Length of stay, median (IQR)	2 (1–2)	1 (1–2)	2 (1–3)	0.1398
**Severity Score**	**No. (%)**			
MVSS, median (IQR)	13 (10–15)	13 (10–15)	13 (10–15)	0.9695
*MVSS*				
0–10 mild	75 (30%)	9 (26%)	66 (30%)	
11–15 moderately severe	116 (46%)	19 (54%)	97 (44%)	
16–20 very severe	47 (19%)	5 (14%)	42 (19%)	
Unable to calculate	15 (6%)	2 (6%)	13 (6%)	

^a^ Probability (*p*)-value was determined by Wilcoxon two-sample test for G8P[8] positive vs. Non-G8P[8] positive. ^b^ Subjects were considered eligible if they were over 6 weeks of age and born before 1 April 2006, based on the approval dates of rotavirus vaccines in the United States.

## Data Availability

For the 29 G8P[8] strains analyzed by NGS, the nucleotide sequences of each gene were deposited in GenBank. Accession numbers are presented in Table S4.

## Supplementary Materials

The following supporting information can be downloaded at: https://www.mdpi.com/article/10.3390/v17091230/s1, Table S1. Nucleotide and amino acid identity percentages for VP7 genes among NVSN strains and between NVSN strains and GenBank sequences, G8 lineages I through VI; Table S2. Nucleotide and amino acid identity percentages for VP4 genes among NVSN strains and between NVSN strains and GenBank sequences, P[8] lineages I through IV; Table S3. Range of nucleotide and amino acid identity for all internal genes between NVSN strains and GenBank sequences; Table S4. Accession numbers for the NVSN strains sequenced in this study. Accession numbers highlighted in orange represent partial gene sequences, which are at least 500 nucleotides in length and contain at least 50% of the open reading frame (ORF). The remaining sequences contain the complete ORF; Figure S1. Maximum likelihood phylogenetic tree illustrating the genetic relationships among the ORF nucleotide sequences of the G8 VP7 gene for 2016-2017 NVSN strains; Figure S2. Maximum likelihood phylogenetic tree illustrating the genetic relationships among the ORF nucleotide sequences of the P[8] VP4 gene for 2016-2017 NVSN strains; Figure S3. Maximum likelihood phylogenetic tree illustrating the genetic relationships among the ORF nucleotide sequences of the VP6 gene for 2016-2017 NVSN strains; Figure S4. Maximum likelihood phylogenetic tree illustrating the genetic relationships among the ORF nucleotide sequences of the VP1 gene for 2016-2017 NVSN strains; Figure S5. Maximum likelihood phylogenetic tree illustrating the genetic relationships among the ORF nucleotide sequences of the VP2 gene for 2016-2017 NVSN strains; Figure S6. Maximum likelihood phylogenetic tree illustrating the genetic relationships among the ORF nucleotide sequences of the VP3 gene for 2016-2017 NVSN strains; Figure S7. Maximum likelihood phylogenetic tree illustrating the genetic relationships among the ORF nucleotide sequences of the NSP1 gene for 2016-2017 NVSN strains; Figure S8. Maximum likelihood phylogenetic tree illustrating the genetic relationships among the ORF nucleotide sequences of the NSP2 gene for 2016-2017 NVSN strains; Figure S9. Maximum likelihood phylogenetic tree illustrating the genetic relationships among the ORF nucleotide sequences of the NSP3 gene for 2016-2017 NVSN strains; Figure S10. Maximum likelihood phylogenetic tree illustrating the genetic relationships among the ORF nucleotide sequences of the NSP4 gene for 2016-2017 NVSN strains; Figure S11. Maximum likelihood phylogenetic tree illustrating the genetic relationships among the ORF nucleotide sequences of the NSP5 gene for 2016-2017 NVSN strains.

## Abbreviations

The following abbreviations are used in this manuscript:

AGE	Acute gastroenteritis
aa	Amino acid
AICc	Corrected Akaike Information Criterion
BLASTN	Basic Local Alignment Search Tool for Nucleotides
BV-BRC	Bacterial and Viral Bioinformatics Resource Center
CDC	Centers for Disease Control and Prevention
cDNA	Complementary DNA
dsRNA	Double-stranded RNA
ED	Emergency department
ESS	Effective Sample Size
HC	Healthy control
MCCT	Maximum Clade Credibility Tree
MCMC	Markov chain Monte-Carlo
ML	Maximum likelihood
MVSS	Modified Vesikari Severity Score
NCBI	National Center for Biotechnology Information
NGS	Next-generation sequencing
NIH	National Institutes of Health
NSP	Non-structural protein
nt	Nucleotide
NVSN	New Vaccine Surveillance Network
ORF	Open reading frame
qRT-PCR	Quantitative real-time reverse transcription polymerase chain reaction
RCWG	Rotavirus Classification Working Group
RNA	Ribonucleic acid
RVA	Group A rotavirus
tMRCA	Time to Most Recent Common Ancestor
VE	Vaccine effectiveness
VP	Viral protein
