# Supplementary material for: Genomic Analysis of Rotavirus G8P[8] Strains Detected in the United States Through Active Surveillance, 2016–2017
Source: Viruses. 2025 Sep 9;17(9):1230. doi: 10.3390/v17091230 (PMC12474418; doi:10.3390/v17091230)
Supplement: Supplementary file 1 [file viruses-17-01230-s001.zip › viruses-3792321-supplementary.pdf]

	NVSN Strains vs. GenBank Sequences						
	NVSN strains	G8-Lineage I	G8-Lineage II	G8-Lineage III	G8-Lineage IV	G8-Lineage V	G8-Lineage VI
Nucleotide identity (%)	99.7-100	83.2-84.7	84.1-86.0	83.8-86.1	90.0-100	84.6-87.8	89.3-89.8
Amino acid identity (%)	99.4-100	92.4-95.4	92.0-94.4	92.9-97.1	92.9-100	91.6-95.7	95.0-95.4

Table S1. Nucleotide and amino acid identity percentages for VP7 genes among NVSN strains and between NVSN strains and GenBank sequences, G8 lineages I through VI.

	NVSN Strains vs. GenBank Sequences				
	NVSN strains	P[8]-Lineage I	P[8]-Lineage II	P[8]-Lineage III	P[8]-Lineage IV
Nucleotide identity (%)	99.8-100	90.2-90.6	92.4-97.7	93.5-100	88.1-88.6
Amino acid identity (%)	99.5-100	93.8-94.3	95.2-96.8	93.9-100	89.7-92.9

Table S2. Nucleotide and amino acid identity percentages for VP4 genes among NVSN strains and between NVSN strains and GenBank sequences, P[8] lineages I through IV.

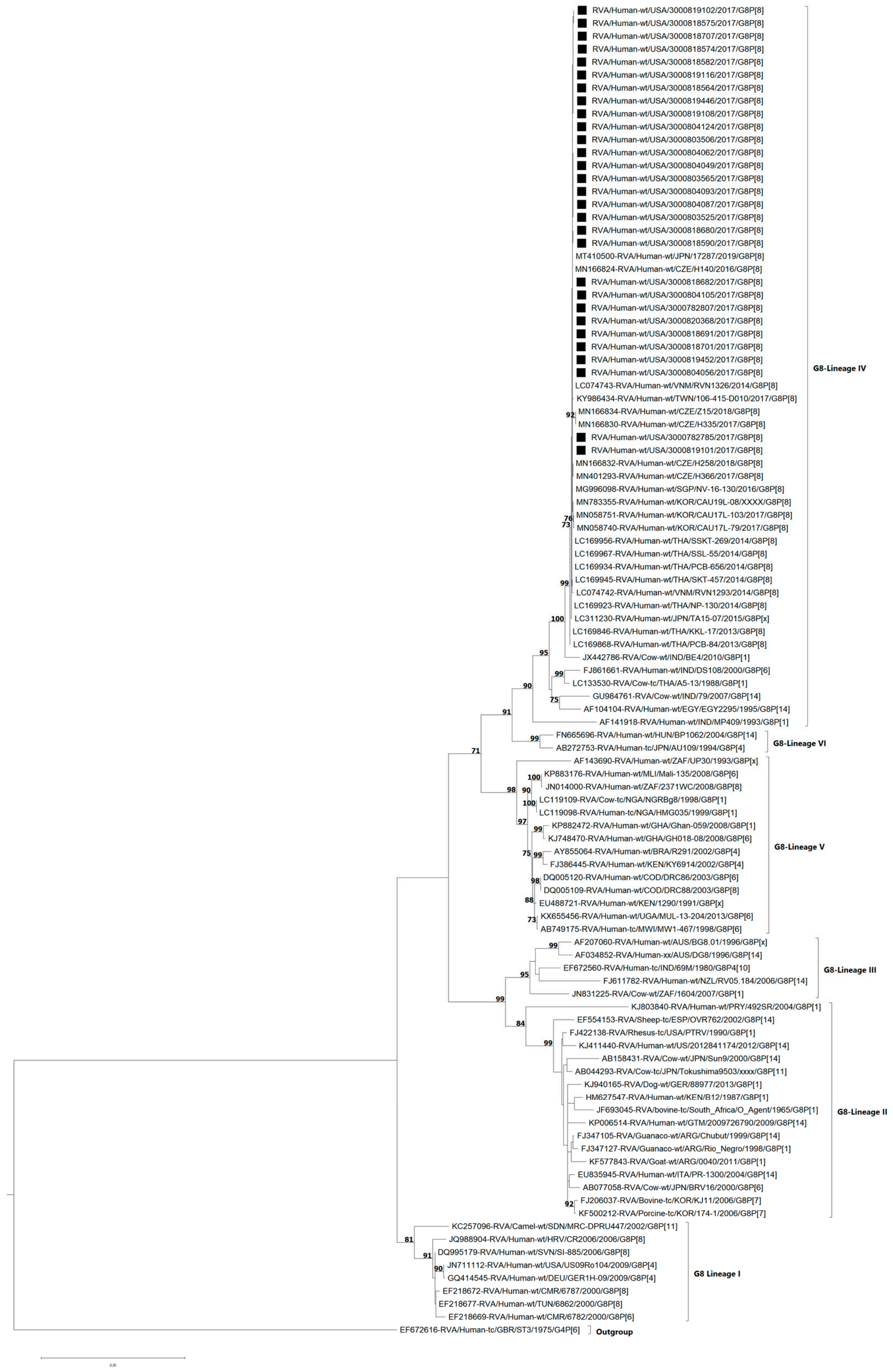

Figure S1. Maximum likelihood phylogenetic tree illustrating the genetic relationships among the ORF nucleotide sequences of the G8 VP7 gene for 2016-2017 NVSN strains. The GTR+G+T evolutionary model was used for phylogenetic inference. NVSN G8P[8] study strains are indicated with a black square. Only bootstrap values ≥70% are shown adjacent to each branch node. Scale bar indicates the number of nucleotide substitutions per site.

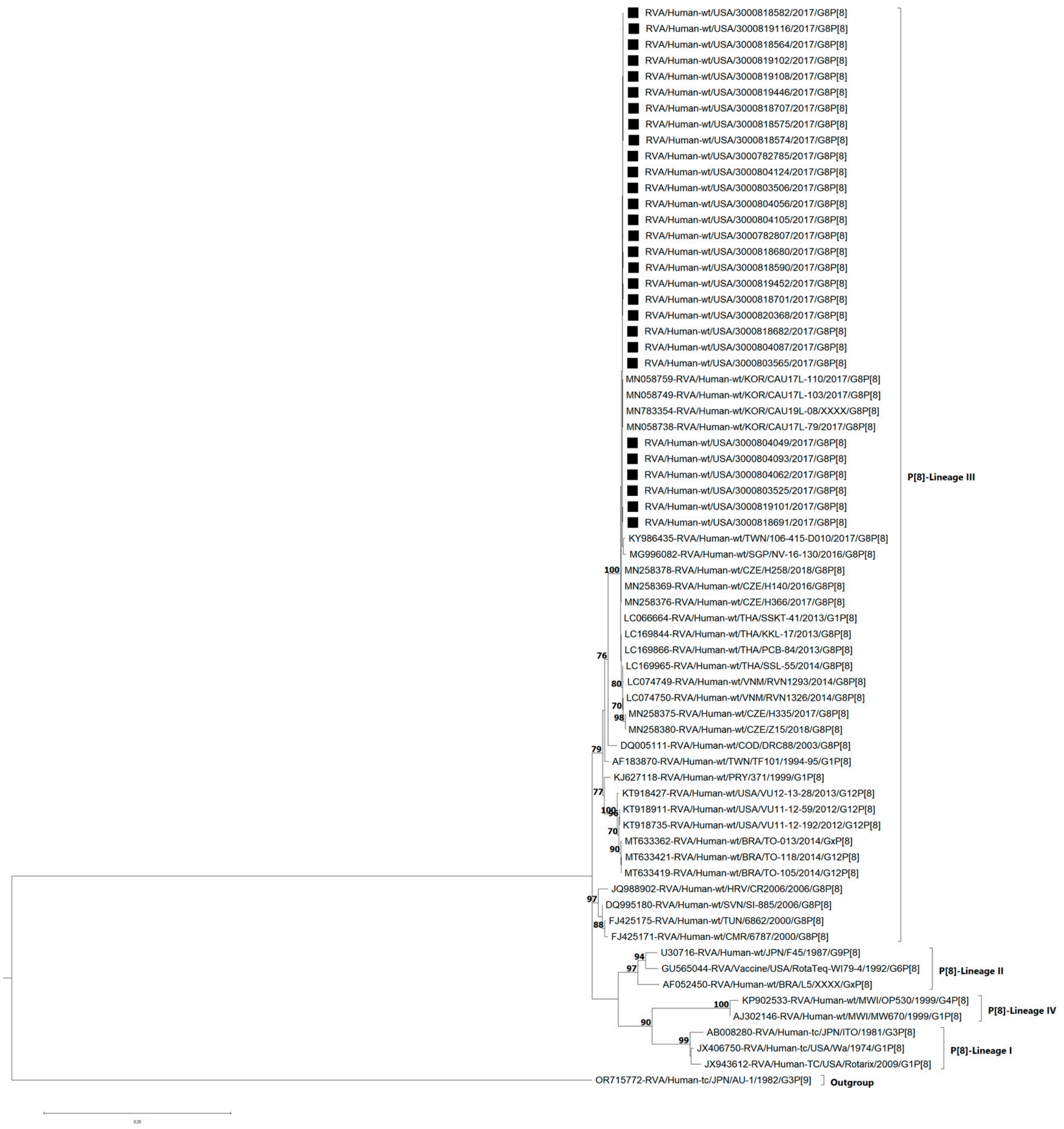

Figure S2. Maximum likelihood phylogenetic tree illustrating the genetic relationships among the ORF nucleotide sequences of the P[8] VP4 gene for 2016-2017 NVSN strains. The GTR+G+T evolutionary model was used for phylogenetic inference. NVSN G8P[8] study strains are indicated with a black square. Lineages are indicated in roman numerals. Only bootstrap values ≥70% are shown adjacent to each branch node. Scale bar indicates the number of nucleotide substitutions per site.

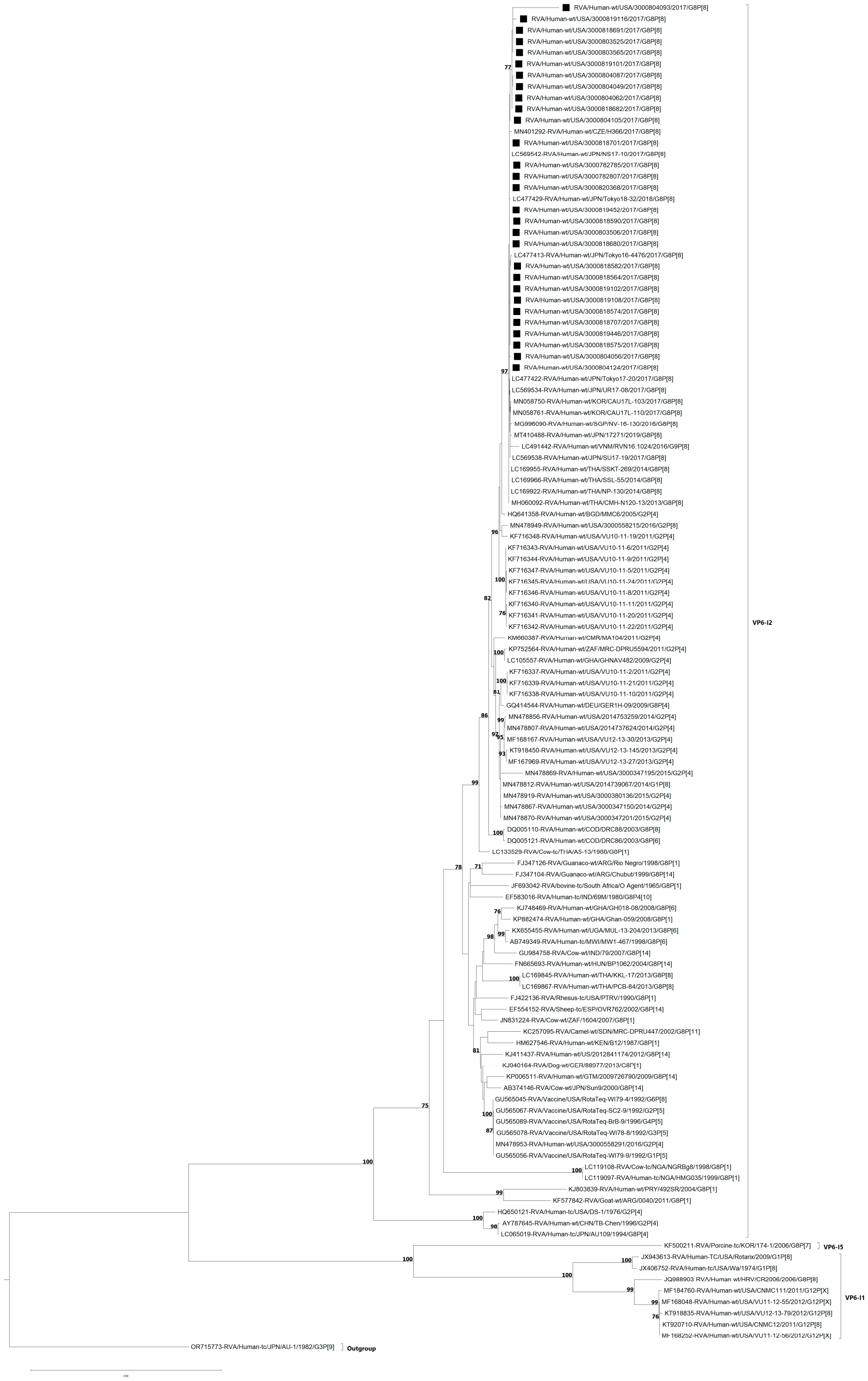

Figure S3. Maximum likelihood phylogenetic tree illustrating the genetic relationships among the ORF nucleotide sequences of the VP6 gene for 2016-2017 NVSN strains. The GTR+G+T evolutionary model was used for phylogenetic inference. NVSN G8P[8] study strains are indicated with a black square. The sequences are annotated with their genotype (I1, I2, and I5) in brackets. Only bootstrap values ≥70% are shown adjacent to each branch node. Scale bar indicates the number of nucleotide substitutions per site. Strain 3000804093 is distinct from the group due to its shorter noncoding 3' end, measuring 25 nt.

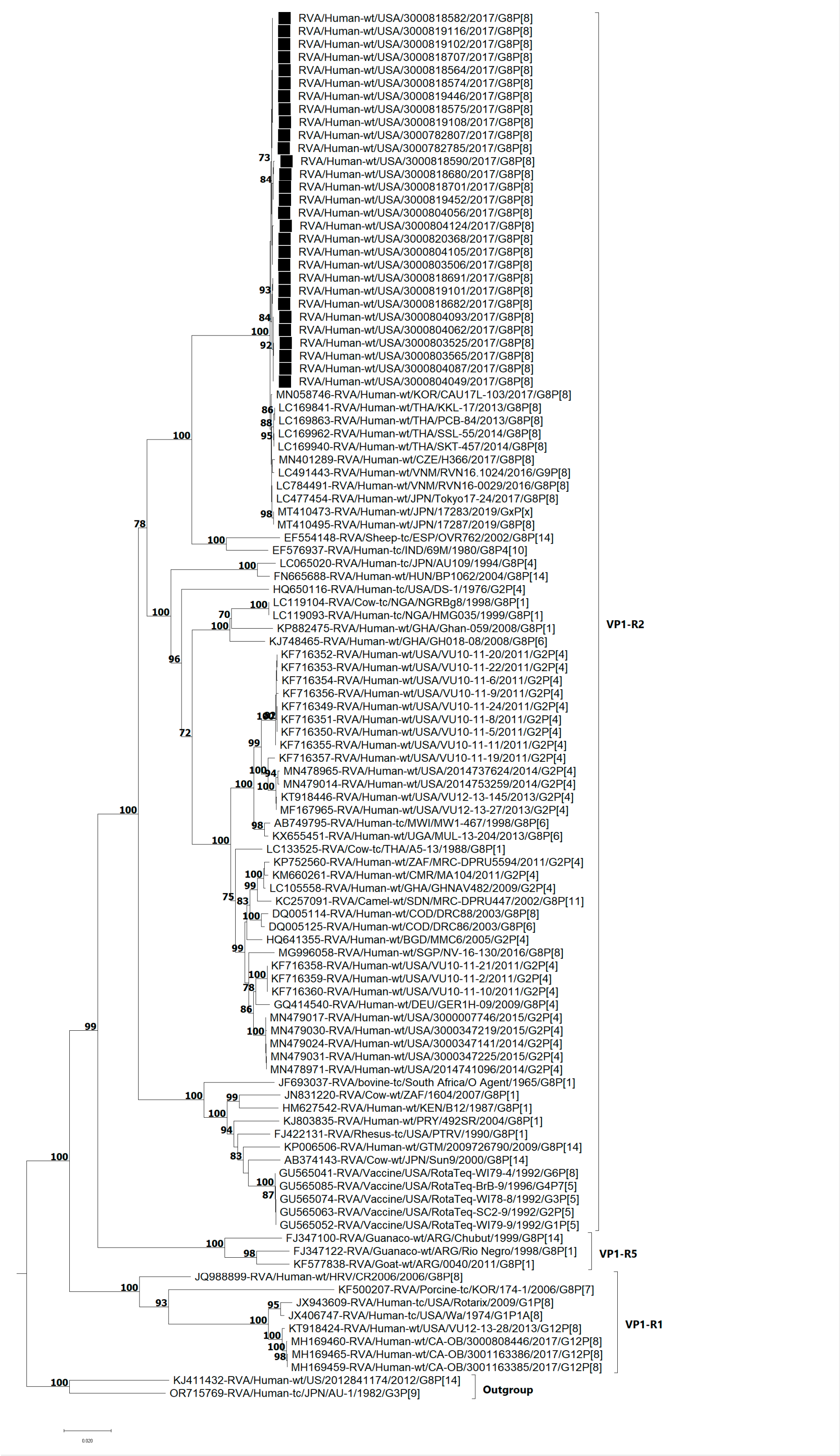

Figure S4. Maximum likelihood phylogenetic tree illustrating the genetic relationships among the ORF nucleotide sequences of the VP1 gene for 2016-2017 NVSN strains. The GTR+G+T evolutionary model was used for phylogenetic inference. NVSN G8P[8] study strains are indicated with a black square. The sequences are annotated with their genotype (R1, R2, and R5) in brackets. Only bootstrap values ≥70% are shown adjacent to each branch node. Scale bar indicates the number of nucleotide substitutions per site.

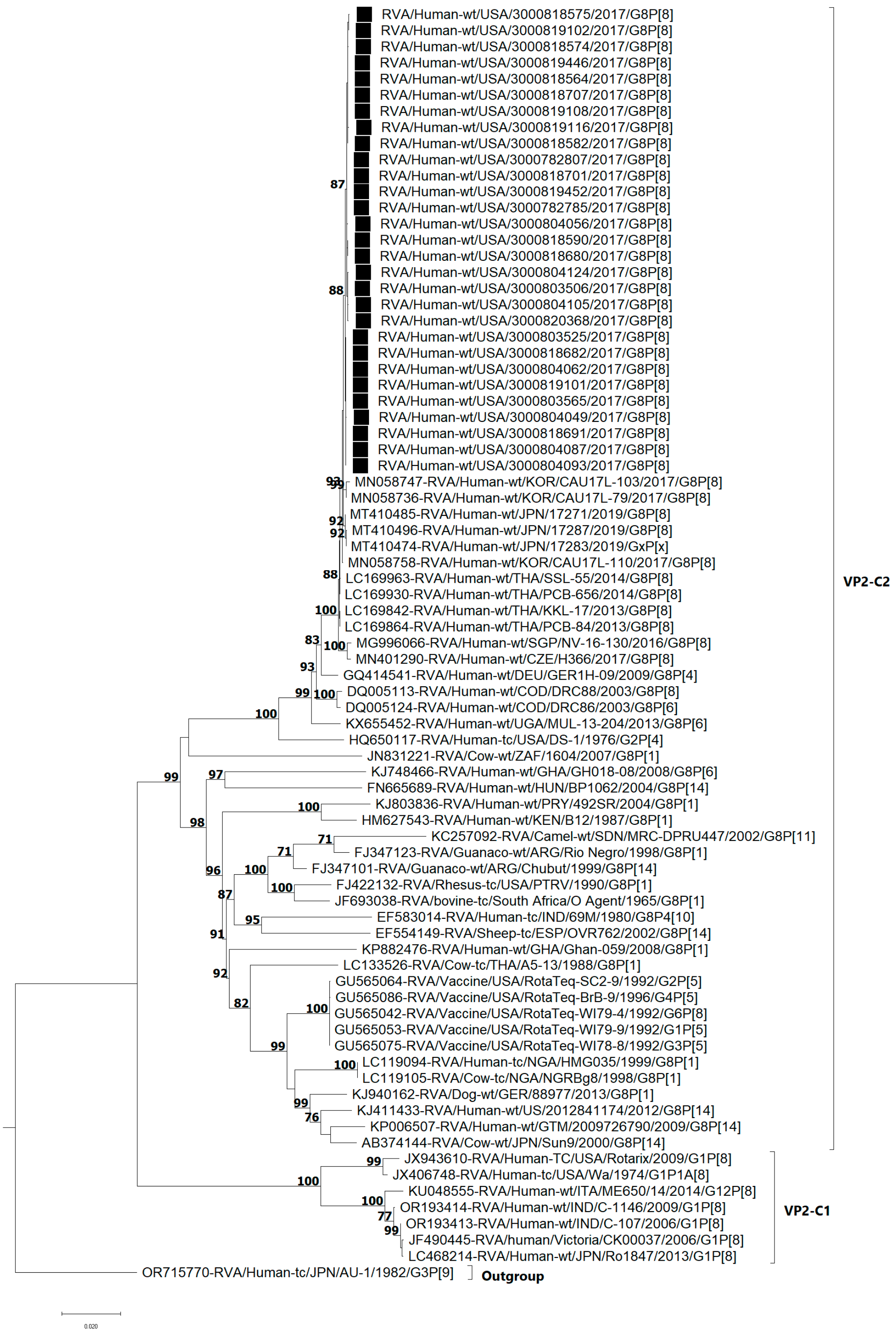

Figure S5. Maximum likelihood phylogenetic tree illustrating the genetic relationships among the ORF nucleotide sequences of the VP2 gene for 2016-2017 NVSN strains. The GTR+G+T evolutionary model was used for phylogenetic inference. NVSN G8P[8] study strains are indicated with a black square. The sequences are annotated with their genotype (C1 and C2) in brackets. Only bootstrap values ≥70% are shown adjacent to each branch node. Scale bar indicates the number of nucleotide substitutions per site.

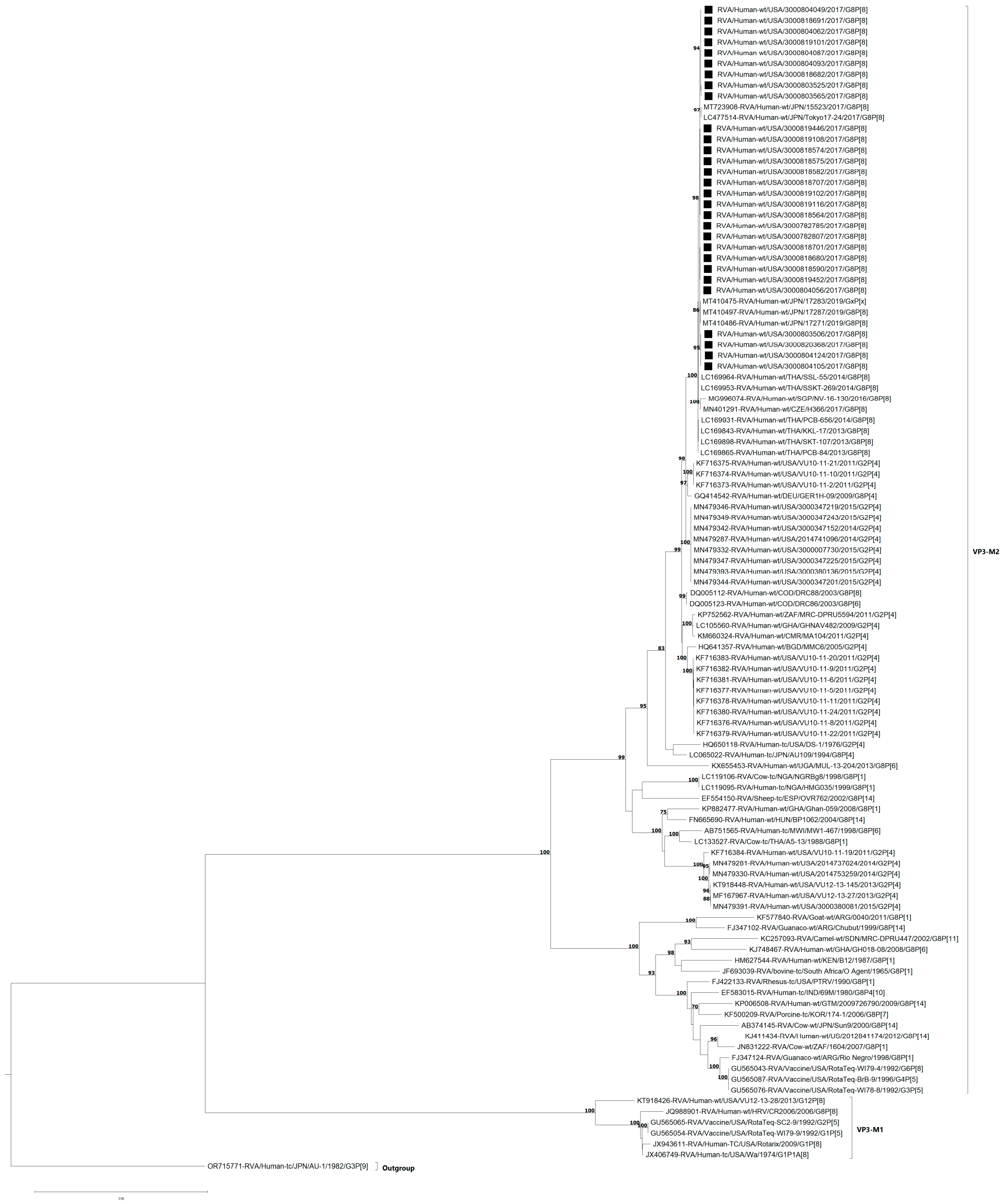

Figure S6. Maximum likelihood phylogenetic tree illustrating the genetic relationships among the ORF nucleotide sequences of the VP3 gene for 2016-2017 NVSN strains. The GTR+G+T evolutionary model was used for phylogenetic inference. NVSN G8P[8] study strains are indicated with a black square. The sequences are annotated with their genotype (M1 and M2) in brackets. Only bootstrap values ≥70% are shown adjacent to each branch node. Scale bar indicates the number of nucleotide substitutions per site.

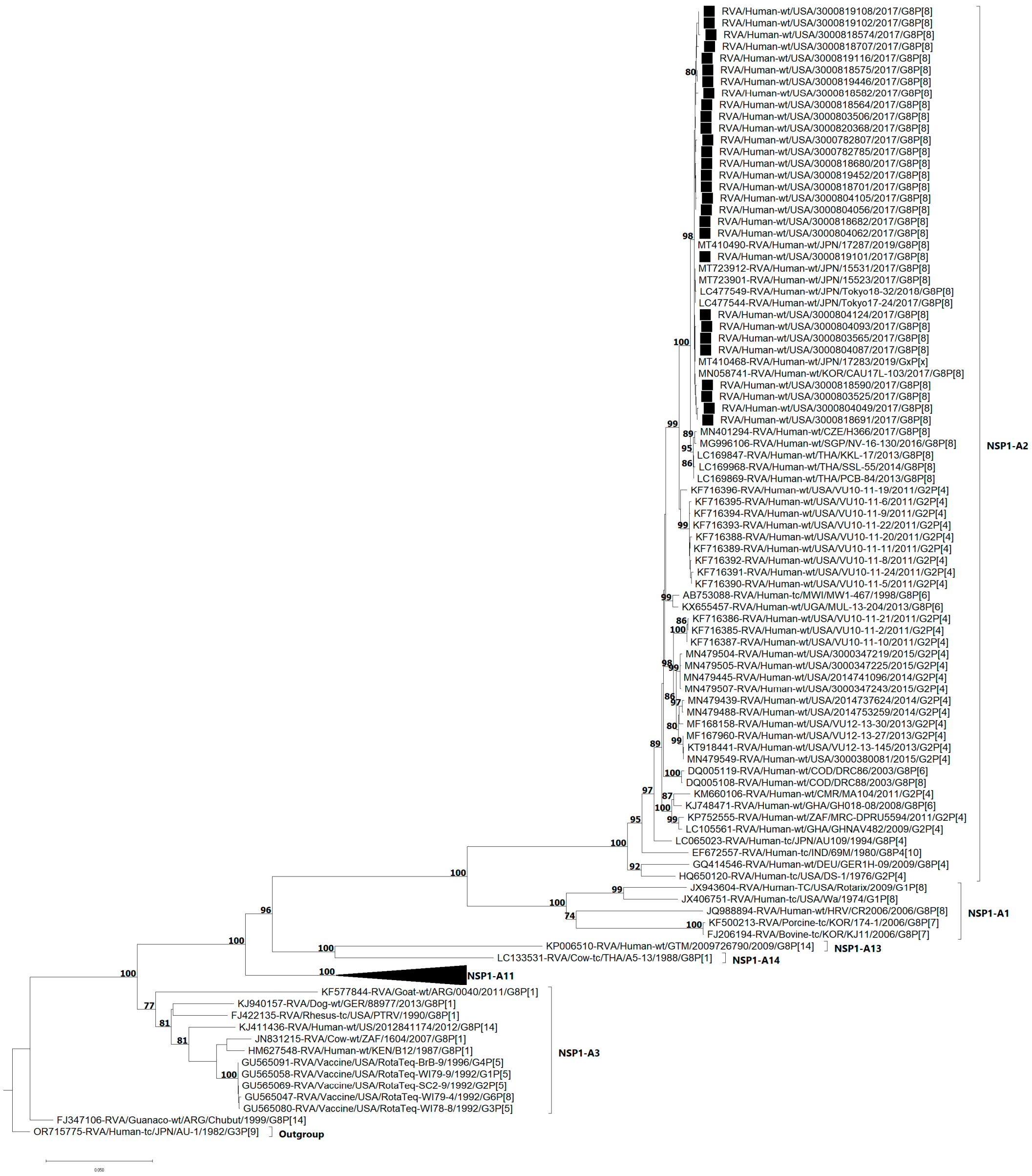

Figure S7. Maximum likelihood phylogenetic tree illustrating the genetic relationships among the ORF nucleotide sequences of the NSP1 gene for 2016-2017 NVSN strains. The GTR+G+T evolutionary model was used for phylogenetic inference. NVSN G8P[8] study strains are indicated with a black square. The sequences are annotated with their genotype (A1, A2, A3, A11, A13, and A14) in brackets. Only bootstrap values ≥70% are shown adjacent to each branch node. Scale bar indicates the number of nucleotide substitutions per site.

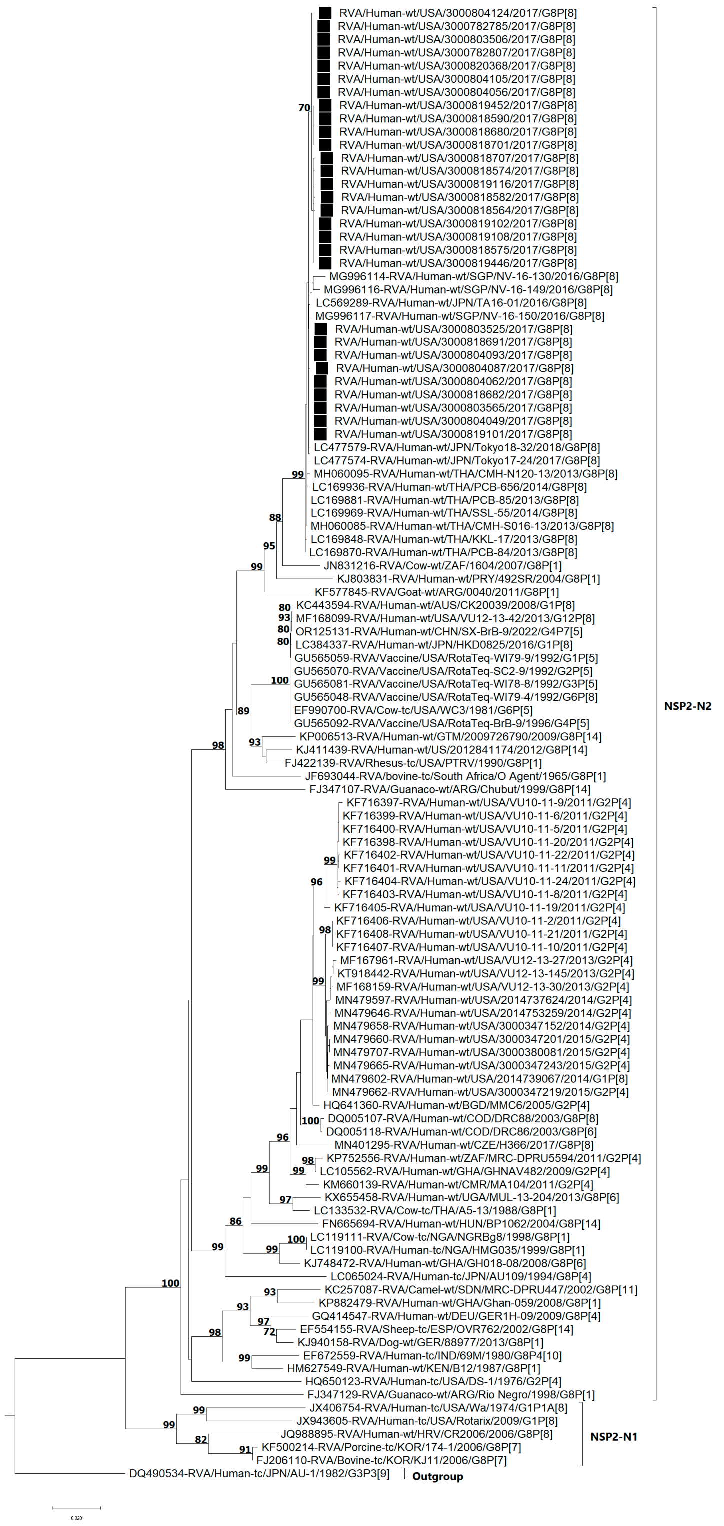

Figure S8. Maximum likelihood phylogenetic tree illustrating the genetic relationships among the ORF nucleotide sequences of the NSP2 gene for 2016-2017 NVSN strains. The GTR+G+T evolutionary model was used for phylogenetic inference. NVSN G8P[8] study strains are indicated with a black square. The sequences are annotated with their genotype (N1 and N2) in brackets. Only bootstrap values ≥70% are shown adjacent to each branch node. Scale bar indicates the number of nucleotide substitutions per site.

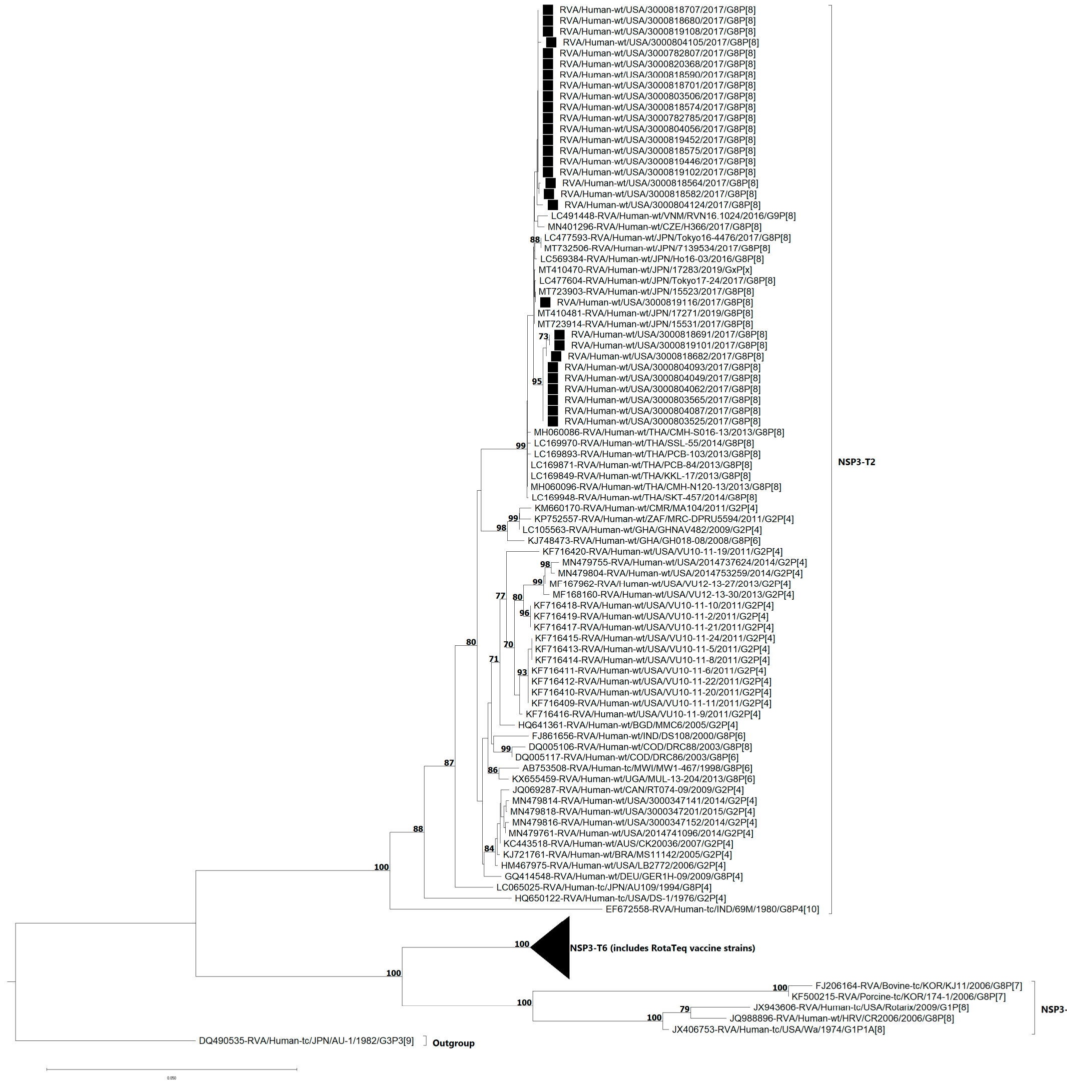

Figure S9. Maximum likelihood phylogenetic tree illustrating the genetic relationships among the ORF nucleotide sequences of the NSP3 gene for 2016-2017 NVSN strains. The GTR+G+T evolutionary model was used for phylogenetic inference. NVSN G8P[8] study strains are indicated with a black square. The sequences are annotated with their genotype (T1, T2, and T6) in brackets. Only bootstrap values ≥70% are shown adjacent to each branch node. Scale bar indicates the number of nucleotide substitutions per site.

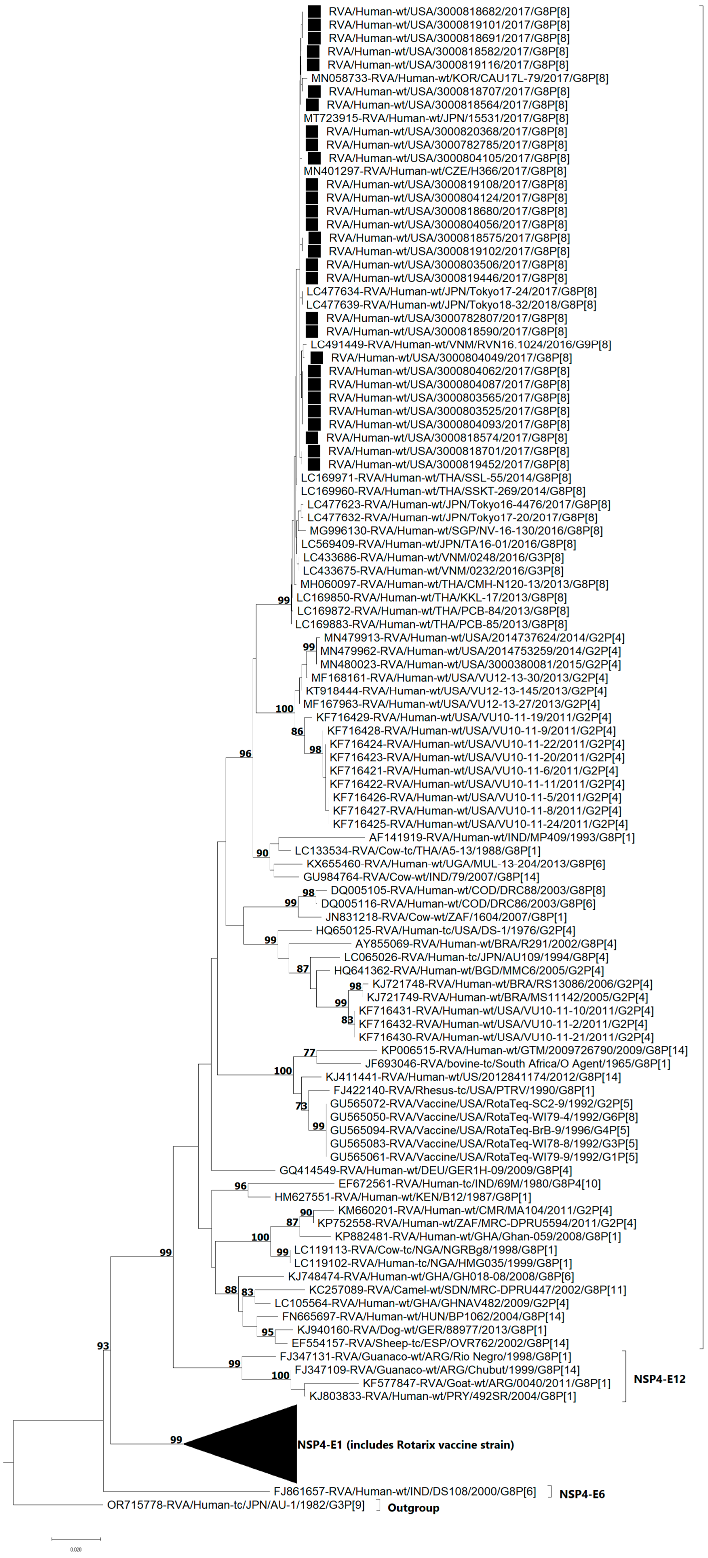

Figure S10. Maximum likelihood phylogenetic tree illustrating the genetic relationships among the ORF nucleotide sequences of the NSP4 gene for 2016-2017 NVSN strains. The GTR+G+T evolutionary model was used for phylogenetic inference. NVSN G8P[8] study strains are indicated with a black square. The sequences are annotated with their genotype (E1, E2, E6, and E12) in brackets. Only bootstrap values ≥70% are shown adjacent to each branch node. Scale bar indicates the number of nucleotide substitutions per site.

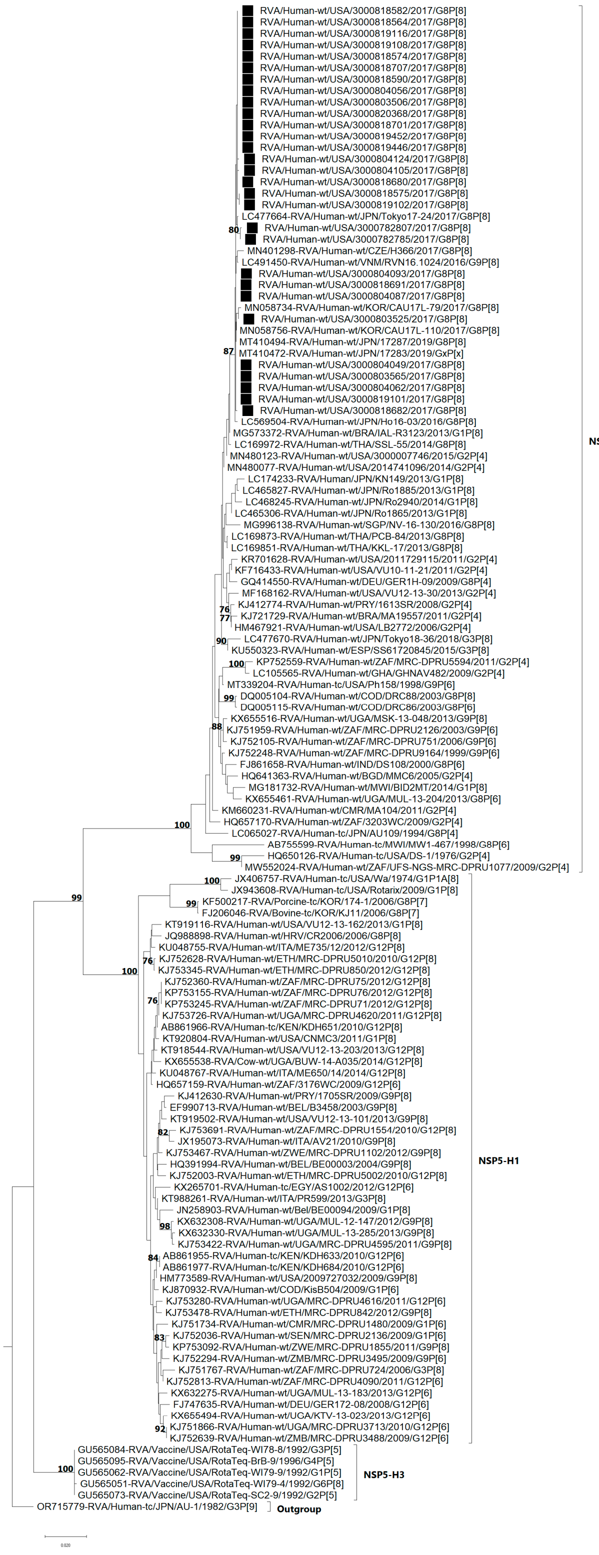

Figure S11. Maximum likelihood phylogenetic tree illustrating the genetic relationships among the ORF nucleotide sequences of the NSP5 gene for 2016-2017 NVSN strains. The GTR+G+T evolutionary model was used for phylogenetic inference. NVSN G8P[8] study strains are indicated with a black square. The sequences are annotated with their genotype (H1, H2, and H3) in brackets. Only bootstrap values ≥70% are shown adjacent to each branch node. Scale bar indicates the number of nucleotide substitutions per site.

	NVSN Strains vs. GenBank Sequences: gene (genotype)								
	**VP6 (I2)**	**VP1 (R2)**	**VP2 (C2)**	**VP3 (M2)**	**NSP1 (A2)**	**NSP2 (N2)**	**NSP3 (T2)**	**NSP4 (E2)**	**NSP5 (H2)**
**Nucleotide identity (%)**	85.5-100	85.6-99.8	85.3-99.8	82.9-99.9	92.3-100	86.5-99.9	90.2-100	87.5-100	95.5-100
**Amino acid identity (%)**	96.5-100	97.0-99.8	96.9-100	89.6-100	91.9-100	94.0-100	94.2-100	94.9-100	96.5-100

Table S3. Range of nucleotide and amino acid identity for all internal genes between NVSN strains and GenBank sequences.

	Strain Name	VP7	VP4	VP6	VP1	VP2	VP3	NSP1	NSP2	NSP3	NSP4	NSP5
**1**	RVA/Human-wt/USA/3000782785/2017/G8P[8]	OP487299	OP487300	OP487301	OP487302	OP487303	OP487304	OP487305	OP487306	OP487307	OP487308	OP487309
**2**	RVA/Human-wt/USA/3000782807/2017/G8P[8]	OP487310	OP487311	OP487312	OP487313	OP487314	OP487315	OP487316	OP487317	OP487318	OP487319	OP487320
**3**	RVA/Human-wt/USA/3000803506/2017/G8P[8]	OP487321	OP487322	OP487323	OP487324	OP487325	OP487326	OP487327	OP487328	OP487329	OP487330	OP487331
**4**	RVA/Human-wt/USA/3000803525/2017/G8P[8]	OP487332	OP487333	OP487334	OP487335	OP487336	OP487337	OP487338	OP487339	OP487340	OP487341	OP487342
**5**	RVA/Human-wt/USA/3000803565/2017/G8P[8]	OP487343	OP487344	OP487345	OP487346	OP487347	OP487348	OP487349	OP487350	OP487351	OP487352	OP487353
**6**	RVA/Human-wt/USA/3000804049/2017/G8P[8]	OP487354	OP487355	OP487356	OP487357	OP487358	OP487359	OP487360	OP487361	OP487362	OP487363	OP487364
**7**	RVA/Human-wt/USA/3000804056/2017/G8P[8]	OP487365	OP487366	OP487367	OP487368	OP487369	OP487370	OP487371	OP487372	OP487373	OP487374	OP487375
**8**	RVA/Human-wt/USA/3000804062/2017/G8P[8]	OP487376	OP487377	OP487378	OP487379	OP487380	OP487381	OP487382	OP487383	OP487384	OP487385	OP487386
**9**	RVA/Human-wt/USA/3000804087/2017/G8P[8]	OP487387	OP487388	OP487389	OP487390	OP487391	OP487392	OP487393	OP487394	OP487395	OP487396	OP487397
**10**	RVA/Human-wt/USA/3000804093/2017/G8P[8]	OP487398	OP487399	OP487400	OP487401	OP487402	OP487403	OP487404	OP487405	OP487406	OP487407	OP487408
**11**	RVA/Human-wt/USA/3000804105/2017/G8P[8]	OP487409	OP487410	OP487411	OP487412	OP487413	OP487414	OP487415	OP487416	OP487417	OP487418	OP487419
**12**	RVA/Human-wt/USA/3000804124/2017/G8P[8]	OP487420	OP487421	OP487422	OP487423	OP487424	OP487425	OP487426	OP487427	OP487428	OP487429	OP487430
**13**	RVA/Human-wt/USA/3000818564/2017/G8P[8]	OR789996	OR789954	OR789975	OR789891	OR789912	OR789933	OR790017	OR790038	OR790059	OR790080	OR790101
**14**	RVA/Human-wt/USA/3000818574/2017/G8P[8]	OR789997	OR789955	OR789976	OR789892	OR789913	OR789934	OR790018	OR790039	OR790060	OR790081	OR790102
**15**	RVA/Human-wt/USA/3000818575/2017/G8P[8]	OR789998	OR789956	OR789977	OR789893	OR789914	OR789935	OR790019	OR790040	OR790061	OR790082	OR790103
**16**	RVA/Human-wt/USA/3000818582/2017/G8P[8]	OR789999	OR789957	OR789978	OR789894	OR789915	OR789936	OR790020	OR790041	OR790062	OR790083	OR790104
**17**	RVA/Human-wt/USA/3000818590/2017/G8P[8]	OR790000	OR789958	OR789979	OR789895	OR789916	OR789937	OR790021	OR790042	OR790063	OR790084	OR790105
**18**	RVA/Human-wt/USA/3000818680/2017/G8P[8]	OR790001	OR789959	OR789980	OR789896	OR789917	OR789938	OR790022	OR790043	OR790064	OR790085	OR790106
**19**	RVA/Human-wt/USA/3000818682/2017/G8P[8]	OR790002	OR789960	OR789981	OR789897	OR789918	OR789939	OR790023	OR790044	OR790065	OR790086	OR790107
**20**	RVA/Human-wt/USA/3000818691/2017/G8P[8]	OR790003	OR789961	OR789982	OR789898	OR789919	OR789940	OR790024	OR790045	OR790066	OR790087	OR790108
**21**	RVA/Human-wt/USA/3000818701/2017/G8P[8]	OR790004	OR789962	OR789983	OR789899	OR789920	OR789941	OR790025	OR790046	OR790067	OR790088	OR790109
**22**	RVA/Human-wt/USA/3000818707/2017/G8P[8]	OR790006	OR789964	OR789985	OR789901	OR789922	OR789943	OR790027	OR790048	OR790069	OR790090	OR790111
**23**	RVA/Human-wt/USA/3000819101/2017/G8P[8]	OR790007	OR789965	OR789986	OR789902	OR789923	OR789944	OR790028	OR790049	OR790070	OR790091	OR790112
**24**	RVA/Human-wt/USA/3000819102/2017/G8P[8]	OR790008	OR789966	OR789987	OR789903	OR789924	OR789945	OR790029	OR790050	OR790071	OR790092	OR790113
**25**	RVA/Human-wt/USA/3000819108/2017/G8P[8]	OR790009	OR789967	OR789988	OR789904	OR789925	OR789946	OR790030	OR790051	OR790072	OR790093	OR790114
**26**	RVA/Human-wt/USA/3000819116/2017/G8P[8]	OR790010	OR789968	OR789989	OR789905	OR789926	OR789947	OR790031	OR790052	OR790073	OR790094	OR790115
**27**	RVA/Human-wt/USA/3000819446/2017/G8P[8]	OP487431	OP487432	OP487433	OP487434	OP487435	OP487436	OP487437	OP487438	OP487439	OP487440	OP487441
**28**	RVA/Human-wt/USA/3000819452/2017/G8P[8]	OR790011	OR789969	OR789990	OR789906	OR789927	OR789948	OR790032	OR790053	OR790074	OR790095	OR790116
**29**	RVA/Human-wt/USA/3000820368/2017/G8P[8]	OR790013	OR789971	OR789992	OR789908	OR789929	OR789950	OR790034	OR790055	OR790076	OR790097	OR790118

Table S4. Accession numbers for the NVSN strains sequenced in this study. Accession numbers highlighted in orange represent partial gene sequences, which are at least 500 nucleotides in length and contain at least 50% of the open reading frame (ORF). The remaining sequences contain the complete ORF.
